# Monitoring metabolic response using FDG PET-CT during targeted therapy for metastatic colorectal cancer

**DOI:** 10.1007/s00259-016-3365-x

**Published:** 2016-04-12

**Authors:** Erwin Woff, Alain Hendlisz, Camilo Garcia, Amelie Deleporte, Thierry Delaunoit, Raphaël Maréchal, Stéphane Holbrechts, Marc Van den Eynde, Gauthier Demolin, Irina Vierasu, Renaud Lhommel, Namur Gauthier, Thomas Guiot, Lieveke Ameye, Patrick Flamen

**Affiliations:** 1 Nuclear Medicine Department, Institut Jules Bordet, Université libre de Bruxelles, 1 rue Héger-Bordet, 1000 Brussels, Belgium; 2 Medical Oncology Department, Institut Jules Bordet, Université libre de Bruxelles, Brussels, Belgium; 3 Oncology Department, Jolimont Hospital, Haine-St-Paul, Belgium; 4 Gastroenterology Medico-Surgical Department, Erasme University Hospital, Université libre de Bruxelles, Brussels, Belgium; 5 Oncology Department, CHU Ambroise Paré, Mons, Belgium; 6 Oncology Department, Cliniques Universitaires Saint-Luc, Université catholique de Louvain, Brussels, Belgium; 7 Gastroenterology Department, CHC Saint-Joseph, Liège, Belgium; 8 Nuclear Medicine Department, Erasme University Hospital, Université libre de Bruxelles, Brussels, Belgium; 9 Nuclear Medicine Department, Cliniques Universitaires Saint-Luc, Université catholique de Louvain, Brussels, Belgium; 10 Nuclear Medicine Department, CHC Saint-Joseph, Liège, Belgium; 11 Data center Department, Institut Jules Bordet, Université libre de Bruxelles, Brussels, Belgium

**Keywords:** Early metabolic response assessment, FDG PET-CT, Tumoral heterogeneity, Metastatic colorectal cancer, Targeted therapy

## Abstract

**Introduction:**

The introduction of targeted drugs has had a significant impact on the approach to assessing tumour response. These drugs often induce a rapid cytostatic effect associated with a less pronounced and slower tumoural volume reduction, thereby impairing the correlation between the absence of tumour shrinkage and the patient’s unlikelihood of benefit. The aim of the study was to assess the predictive value of early metabolic response (mR) evaluation after one cycle, and its interlesional heterogeneity to a later metabolic and morphological response assessment performed after three cycles in metastatic colorectal cancer (mCRC) patients treated with combined sorafenib and capecitabine.

**Methods:**

This substudy was performed within the framework of a wider prospective multicenter study on the predictive value of early FDG PET-CT response assessment (SoMore study). A lesion-based response analysis was performed, including all measurable lesions identified on the baseline PET. On a per-patient basis, a descriptive 4-class response categorization was applied based upon the presence and proportion of non-responding lesions. For dichotomic response comparison, all patients with at least one resistant lesion were classified as non-responding.

**Results:**

On baseline FDG PET-CT, 124 measurable “target” lesions were identified in 38 patients. Early mR assessments showed 18 patients (47 %) without treatment resistant lesions and 12 patients (32 %) with interlesional response heterogeneity. The NPV and PPV of early mR were 85 % (35/41) and 84 % (70/83), respectively, on a per-lesion basis and 95 % (19/20) and 72 % (13/18), respectively, on a dichotomized per-patient basis.

**Conclusions:**

Early mR assessment performed after one cycle of sorafenib-capecitabine in mCRC is highly predictive of non-response at a standard response assessment time. The high NPV (95 %) of early mR could be useful as the basis for early treatment discontinuation or adaptation to spare patients from exposure to non-effective drugs.

## Introduction

Colorectal cancer is the third leading cause of cancer death in the world and continues to be a major health problem worldwide [1]. Despite improvements in chemotherapy, patients with metastatic colorectal cancer (mCRC) carry a bleak prognosis with less than 10 % survivors at 5 years [1]. Recent improvements have been made thanks to the development of a new generation of oral multikinases inhibitors interfering with cellular proliferation, tumour growth, and angiogenesis [2]. Most of these drugs are only effective in a limited number of patients and are associated with significant drug-related morbidity and a high economic impact for the society [3, 4]. Therefore, it becomes essential to improve the cost-effectiveness of these new drug regimens through the development of predictive biomarkers designed for a rapid identification of non-responding patients.

 These new medications induce a rare and slow tumoural shrinkage. Standard RECIST criteria-based morphological response assessment is often limited by its low sensitivity for detecting objective response [5]. Early data indicate that a sensitive response detection using FDG PET-CT performed as soon as after one cycle of treatment is possible, and is correlated with treatment outcome, mostly due to the early identification of non-responding patients [6–10]. This justifies studies about the use of FDG PET-CT for metabolic response (mR) assessment [4, 11–14].

An imaging biomarker for early non-response detection should have a high negative predictive value to avoid unjustified early discontinuation of an effective treatment. False-negative early responses should be avoided through the use of a low response cut-off, with a decrease of FDG uptake (expressed as SUV_max_) of less than 15 % compared to baseline [15].

Highlighting the interlesional heterogeneity of mR at an early time point during treatment could be of utmost importance for the prediction of a patient’s outcome and for clinical management. Rapid identification and localization of resistant lesion(s) could be the basis for a more individualized treatment approach.

The objective of this substudy was to assess the evolution of metabolic resistance over time during therapy.

## Materials and methods

### Study design

This substudy was performed within the framework of a wider prospective phase II multicenter study on the predictive value of FDG PET-CT response assessment for patient outcome predictions in patients with mCRC treated with a combination of sorafenib and capecitabine (EudraCT number: 2010-023695-91; SoMore study clinical trials NCT01290926) [14, 16].

This substudy was conducted at four PET-CT centers, in collaboration with six clinical participating institutions, in which patients underwent an early PET at week 3 and an additional “late PET” at week 6–8 (range: 38–62 days) together with the standard RECIST-based morphological response assessment.

**Fig. 1:**
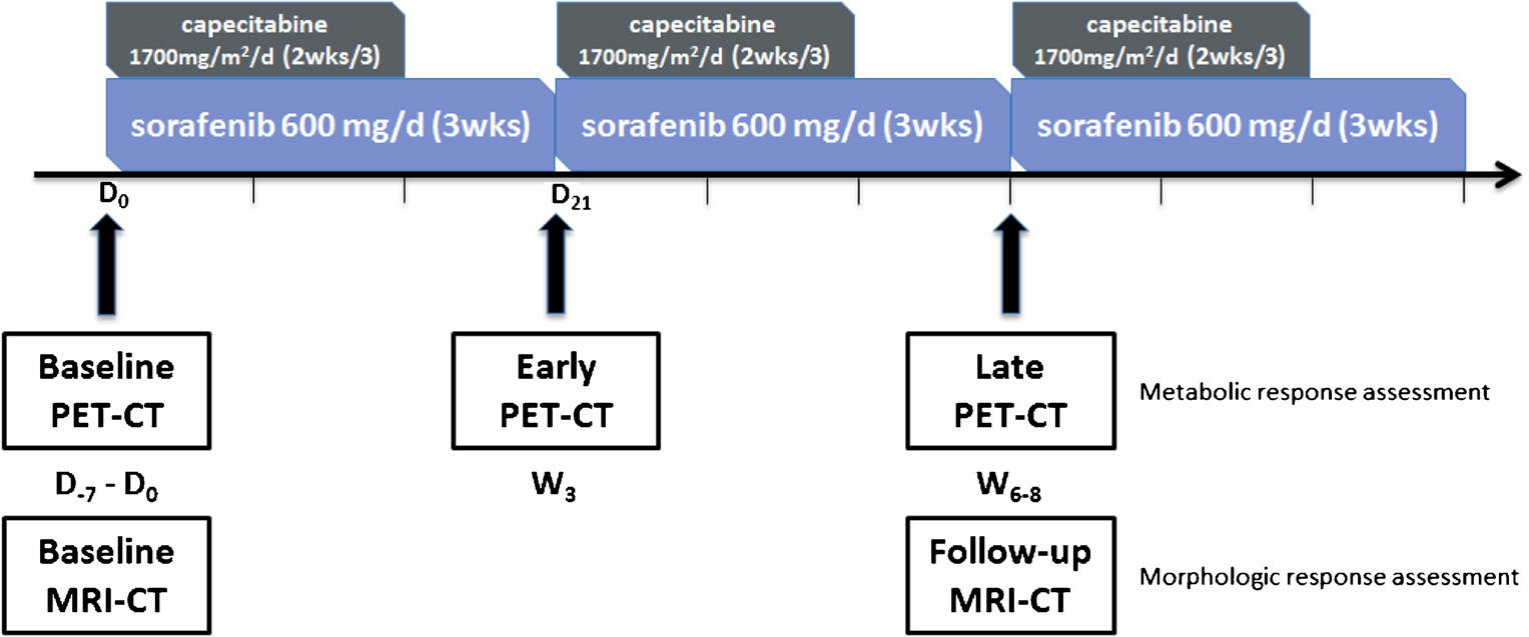
Illustration of the study design. D-7 - D0: day −7 until day 0; W3: week 3; W6-8: weeks 6–8

A diagram of the study design is shown in Fig. 1.

The early PET assessment was blinded to the referring oncologists, and did not interfere with treatment decisions. This substudy was approved by the Jules Bordet Institute Ethics Committee (CE2176) and Ethics Committees of all other participating centers.

### Patients

From the total cohort of 97 patients included in the SoMore trial, 41 patients were prospectively enrolled in this substudy. The patients’ characteristics are shown in Table 1. Enrollment criteria were: age > 18; Eastern Cooperative Oncology Group (ECOG) performance status ≤ 1; life expectancy > 12 weeks; at least one RECIST evaluable lesion; ability to undergo the therapy, and signed informed consent [17]. Patients received 600 mg of sorafenib with escalation to 800 mg, if well tolerated. The study dose of capecitabine was 850 mg/m^2^ twice daily for 14 days, followed by a 7-day rest period. Changes in therapy other than dose adaptation for toxicity were not allowed. Treatment stopping rules were defined according to either excessive toxicity or clinical or radiological progression.

**Table 1:** Characteristics of patients. ECOG: eastern cooperative oncology group

Characteristics	*n*	%
Number	38	
Age		
Mean ± SD	62 ± 10	
Median (min-max)	64 (41 – 76)	
Gender		
Men	22	58 %
Women	16	42 %
ECOG Performance Status		
0	23	61 %
1	15	39 %
Previous use of Bevacizumab		
No	20	53 %
Yes	18	47 %
Number of previous chemotherapy lines		
2	18	47 %
3	12	32 %
4	3	8 %
5	2	5 %
6	3	8 %

### FDG PET-CT procedures

All patients were studied using a dedicated FDG PET-CT camera of the locally available brand. Most of the FDG PET-CT images (92 %) were acquired at the Jules Bordet Institute using a General Electric (GE) Discovery 690 time of flight (TOF) PET system, 60 min after injection (range: 60–70 min and did not differ by more than 15 min from the uptake time for the baseline FDG PET-CT) of 3.7–4 MBq/kg. All PET scans were acquired in three-dimensional mode with an acquisition time of 90 s per bed position with an overlap of 23.4 %. PET images were reconstructed with the built-in GE VUE Point Fx algorithm, an ordered subset expectation maximization algorithm with two iterations and 18 subsets, and were postfiltered with a 6.4-mm full-width at half-maximum (FWHM) Gaussian function. The images were corrected for attenuation and for scatter using the CT data. CT was performed with 64 slices helical scanner (VCT; GE Medical Systems). The tension was 120 kV, and the current was modulated by the Auto-mA software with a noise index of 30 (range: 30–200 mA) and ASIR®. The other CT acquisition parameters were 0.5 s per CT rotation and a pitch of 0.98. The CT images were reconstructed with the ASIR algorithm set at 40 %, with a matrix of 512 × 512 (0.97 × 0.97 mm pixel size) and a slice thickness of 2.5 mm. The PET matrix was 192 × 192 pixels of 2.73 × 2.73 mm with a slice thickness of 3.27 mm.

For three patients, FDG PET-CT images were acquired using a Philips Gemini 16P (for one patient), a Philips Gemini TOF 16 (for one patient), and a GE Discovery LS system (for one patient). Patient preparation (>6 h fasting, blood glucose levels less than 150 mg/dL before FDG injection), imaging, and reconstruction protocols were kept constant for all FDG PET-CT performed in the four standardized PET-CT centers.

The time interval between tracer injection and start of the PET-CT acquisition at early and late time points did not differ by more than 15 min from the interval recorded at baseline. The difference of net injected activity between the baseline and the two subsequent scans did not exceed 20 %, except for one patient (difference of −47 % compared to baseline).

### FDG PET-CT analysis

All FDG PET-CT images were independently reviewed by two experienced nuclear medicine physicians not involved in treating the patients and blinded to both medical records and treatment outcomes on a dedicated workstation (Advantage Workstation; GE Healthcare) using the commercial PET VCAR software 4.6. The mR assessment process comprised four phases: identifying the measurable (target) lesions on the baseline FDG PET-CT; assessing the mR of each target lesion (lesion-based mR); categorizing the mR distribution into four classes; and dichotomizing the overall mR (patient-based mR).

Both reviews were compared. In all cases of mR classification discrepancy, a final unanimous consensus was achieved.

*Criteria for identification of target lesions:* The criteria were adapted from PERCIST [18]. At baseline, FDG PET-CT target lesions were defined as follows: lesion size >15 mm in transversal diameter on a registered CT image, and a marked accumulation of FDG with SUV normalized to lean body mass higher than 1.5 x mean liver SUV + 2 x SD of mean liver SUV or, in the presence of liver metastasis, 2.0 x mean aorta SUV + 3 x SD of mean aorta SUV. Normal background FDG uptake was determined by drawing a reference area as a 3 cm diameter spherical region of interest (ROI) in the right lobe of the liver. In patients with liver metastases, the reference area was drawn as a 2 cm diameter spherical ROI in the descending thoracic aorta. The maximal number of target lesions was non-restricted.

*Lesion-based mR assessment:* This assessment was performed on both early and late PET-CTs, and the defined response to therapy for each target lesion was expressed as a continuous variable representing the percentage change in SUV_max_ between the baseline PET and early/late PET according to the following formula: delta SUVmax = (SUV_max_ response – SUV_max_ baseline)/SUV_max_ baseline. Early mR were classified by applying a response threshold of a 15 % decrease of SUV_max_. Such a low cut-off was chosen to obtain the highest negative predictive value for response, as calculated by Buvat et al. [15]. Late mR performed after three cycles was defined using the EORTC criteria for PET-response assessment and had a cut-off value of a 25 % decrease [19]. Progressive metabolic disease (mPD) was defined as an increase of at least 25 % in SUV_max_ for the early and late mR assessments, or the appearance of a new FDG-avid metastatic lesion. For both time points, a complete metabolic response (mCR) was considered as a complete resolution of FDG uptake within a measurable target lesion to a level less than or equal to that of mean liver activity. A stable metabolic disease (mSD) was between partial metabolic response (mPR) and mPD. For the lesion-based dichotomic response analysis, mPR or mCR lesions were classified as responding lesions (mR), whereas mSD or mPD lesions were classified as non-responding lesions (mNR).

**Fig. 2:**
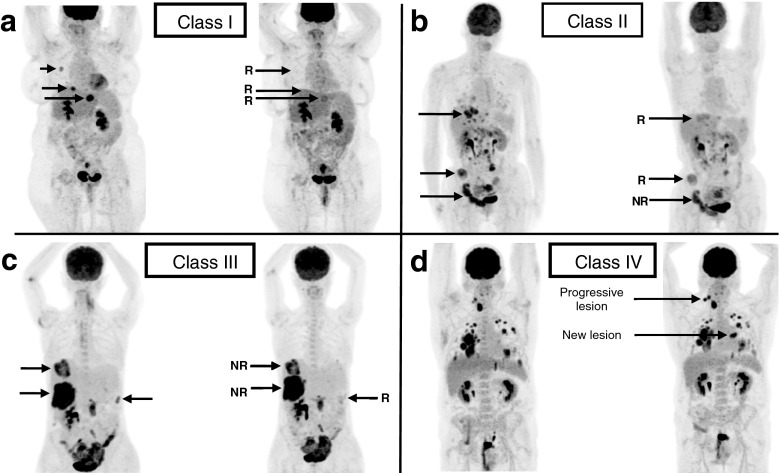
Representative examples of each metabolic dominance response classification. **a** Class I: absence of non-responding lesions **b** Class II: mixed response with a minor proportion of non-responding tumour load **c** Class III: mixed response with a major proportion of non-responding tumour load** d** Class IV: all lesions showed non-response, or the presence of at least one progressive lesion or the appearance of a new lesion

*Patient-based mR assessment:* To describe interlesional response heterogeneity on early PET and its evolution on late PET response, a previously described descriptive method was used [6]. Based on the results of the lesion-based semi-quantitative analysis, patients were grouped into four classes: class I (absence of non-responding lesions), class II (mixed response, minor proportion of tumour load is non-responding), class III (mixed response, major proportion of tumour load is non-responding), and class IV (all lesions showed non-response, or presence of at least one progressive lesion, or appearance of a new lesion). Figure 2 shows examples of each mR class.

*Patient-based mR dichotomization:* For response dichotomization, based on the results of the primary study where response classes have been correlated with patient outcome parameters (OS/PFS), a patient was classified as a responder when there was an absence of metabolically refractory lesions [6]. Therefore, were considered as metabolic responders, all patients showing no metabolic treatment resistant lesion (class I) and as metabolic non-responders, all patients showing a metabolic heterogeneous response (classes II, III, and IV).

### Statistical analysis

Several reports have found a correlation between late mR and patient outcome; therefore, late mR was considered to be a surrogate endpoint for the current analysis [10, 20]. The hypothesis of this substudy was that by using serial FDG PET-CT, the presence and proportion of lesions resistant to treatment can already be identified after one treatment cycle. Therefore, early mR was compared to the standard “late” mR on both a per-lesion and a per-patient basis.

To calculate the predictive values (positive predictive value (PPV), negative predictive value (NPV)), and the concordance rates of early mR on late mR, we constructed 2 × 2 contingency tables for both patient-based and lesion-based analyses. The early and late mR were outlined in all contingency tables. Sensitivity, specificity, PPV, and NPV were calculated with 95 % CIs based on Wilson’s method. The Kaplan–Meier product limit method was used to describe progression-free survival (PFS) and overall survival (OS) curves. The log-rank test was applied to test the statistical significance of differences between survival curves of early mR patients with a homogeneous response (class I) and non-responder patients with or without a heterogeneous response (classes II, III, and IV). To evaluate a potential selection bias for patients included in this substudy, the difference between the survival curves of patients included in the primary study and in this substudy was checked.

## Results

### Evaluable population

A total of 41 patients were included in the current study. Three patients were excluded from the mR analysis due to a lack of target lesions identified on the baseline PET-CT for one patient and a major violation of the standardized imaging procedure for two other patients. Among the seven clinical centers participating in the primary trial, three centers systematically performed a late mR assessment (corresponding to 35/38 (92 %) included patients), while three other centers only sporadically performed a late PET (corresponding to 6/37 (16 %) included patients) and one center, which did not perform a late PET (corresponding to 0/7 included patients).

Disease involvement was observed in the liver (*n* = 47), lungs (*n* = 26), lymph nodes (*n* = 24), bones (*n* = 7), peritoneum (*n* = 6) (carcinomatosis), large bowel (*n* = 3), pancreas (*n* = 3), rectum (*n* = 2), sigmoid (*n* = 2), adrenals (*n* = 2), muscle (*n* = 1), and pleura (*n* = 1) (Table 2).

**Table 3:** Agreement on lesion-based mR between early and late PET

Metabolic response		Late PET				
		mCR	mPR	mSD	mPD	Total
Early PET	mCR	5	0	0	0	5
	mPR	10	55	13	0	78
	mSD	0	6	20	1	27
	mPD	0	0	4	10	14
Total		15	61	37	11	124

### Lesion-based early mR prediction

At the lesion-based level, a total of 124 lesions were analysed. Early mNR was predictive of the late mNR, with a NPV of 35/41 (85 %) lesions. Presence of early mR was predictive of the late mR, with a PPV of 70/83 (84 %) lesions. The concordance rates between early and late mR classes was 73 % with the four-class classification and 85 % with the dichotomized mR (Tables 3 and 4a).

**Table 4:** Agreement between early and late PET with dichotomization of response for lesion-based mR (A) and patient-based mR (B)

a) Lesions were considered as responding if they were classified as mCR or mPR and as non-responding if they were classified as mSD or mPD						
Late PET						
		R	NR	Total	Sensitivity	0.92
	R	70	13	83	Specificity	0.73
Early PET	NR	6	35	41	PPV	0.84
	Total	76	48	124	NPV	0.85
b) Patients were considered responders if they were classified in class I and non-responders if they were classified in classes II, III or IV, according to metabolic dominance response criteria						
Late PET						
		R	NR	Total	Sensitivity	0.93
	R	13	5	18	Specificity	0.79
Early PET	NR	1	19	20	PPV	0.72
	Total	14	24	38	NPV	0.95

**Table 5:** Agreement on patient-based mR between early and late PET according to the four classes of metabolic dominance response criteria

Metabolic response		Late PET				
		I	II	III	IV	Total
Early PET	I	13	4	0	1	18
	II	1	5	2	1	9
	III	0	0	2	1	3
	IV	0	0	0	8	8
Total		14	9	4	11	38

**Fig. 3:**
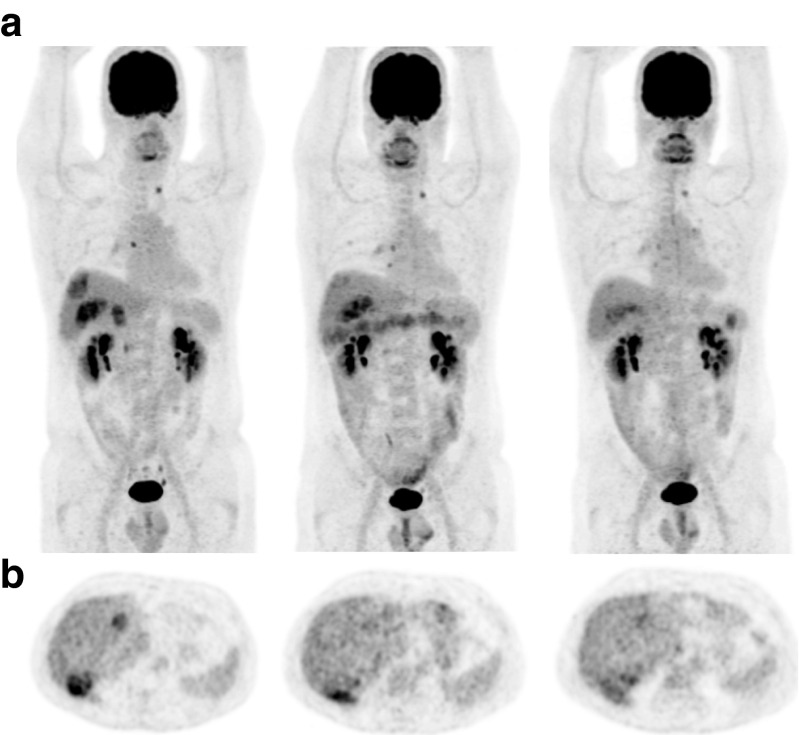
Illustration of the metabolic discordant response between early and late PET evaluations. **a** Patient-based discordant mR: baseline coronal maximum intensity projection (MIP) showing two highly metabolic right hepatic lesions (*left*), week 3 MIP mixed mR of hepatic target lesions (class II) (*middle*), and week 6 MIP homogenous mR of all hepatic target lesions (class I) (*right*). **b** Lesion-based discordant mR: baseline axial PET slice showing the two highly metabolic right hepatic lesions (*left*), week 3 PET mSD of the right posterior hepatic lesion and mCR of the hepatic dome lesion (*middle*), and week 6 PET mPR of the right posterior hepatic lesion (*right*)

*False-negative early mR*: Only 6/124 (5 %) lesions did not respond at the early metabolic evaluation but did respond at the late one. These six lesions were all classified as mSD in the early evaluation and as mPR in the late one. The relative decrease of the metabolism of 4/6 lesions was close to the cut-off for early and late PET (delta SUVmax: −14.3, −14.2, −14.5 and −14.7 % for early PET; delta SUVmax: −26.5, −25.8, −25.2 and −26.3 % for late PET, respectively). The analyses of the two remaining lesions revealed a substantial decrease in their metabolism (delta SUVmax: −28.8 and −27.5 %) between the early (delta SUVmax: −4.9 and −11.2 %) and late (delta SUVmax: −33.7 and −38.7 %) metabolic evaluations. These two lesions were found in two different patients. The first lesion, located in the liver, was the only one that responded at the late metabolic evaluation (delta SUVmax: −33.7 %) compared to the other mNR target lesions in this patient. The second lesion, also located in liver, showed an early mSD (delta SUVmax: −11.2 %) and a late mPR (delta SUVmax: −38.7 %), while the only other target lesion showed an early mPR (delta SUVmax: −30.6 %) and a late mCR (delta SUVmax: −35.5 %). These two false-negative early responding lesions showed a decrease in their metabolism at the early PET, but the decrease was not sufficient to reach the cut-off value, fixed at −15 % of SUV_max_. An illustration of this metabolic discordant response between the early and late PET evaluations of the second lesion is shown in Fig. 3. It is worth noting that an increase of target lesion metabolism (>15 % of SUV_max_) at the early metabolic evaluation followed by a decrease at the late one (so-called “metabolic flare”) was never observed in our study.

*False-positive early mR*: A total of 13/124 (10 %) lesions did respond at the early PET but did not respond at the late PET (Table 4a). These 13 lesions were classified as mPR for the early and mSD for the late PET evaluation (Table 3). Two lesions (in two patients) had a relative decrease of the lesion’s metabolism but not reaching the cut-off value for late mR (delta SUVmax: −24.6 % and −21.3 %). These two lesions were located in the livers of two different patients. All other lesions (*n* = 11, in nine patients) did not show a significant decrease in their metabolism on the late PET compared to the early PET.

### Patient-based early mR prediction

*Interlesional response heterogeneity*: The agreement on patient-based mR between early and late PET according to the four classes of metabolic dominance response criteria is shown in Table 5. The overall concordance rate was 28/38 (74 %).

**Table 2:** Sites of disease involvement and the number and frequency of lesions involved in each site (*N* = 124 lesions)

Sites of disease involvement	Number of lesions	Relative number of lesions (%)
Rectum	2	2
Sigmoid	2	2
Large bowel	3	2
Liver	47	38
Lungs	26	21
Lymph nodes	24	19
Bones	7	6
Peritoneum (carcinomatosis)	6	5
Pancreas	3	2
Adrenals	2	2
Muscle	1	1
Pleura	1	1

*Evolution of response heterogeneity*: All discordances between early and late classifications were observed because patients developed non-responding (resistant) lesions between the early and late mR evaluations. Only one patient out of 38 (3 %) evolved in the opposite way and was classified as Class II at the early metabolic evaluation and Class I at the late one (Table 5). This discordant patient-based response was based on one hepatic lesion described previously that showed an mSD (delta SUVmax: −11.2 %) at the early PET and an mPR (delta SUVmax: −38.7 %) at the late PET, while all the other lesions responded to treatment. Figure 3 illustrates this example.

*Dichotomized patient-based mR*: The concordance rate between early and late PET was 32/38 (84 %) (Table 4b). The metabolic non-response rate was 20/38 (53 %) at the early PET and 24/38 (63 %) at the late PET evaluation (Table 4b). NPV and PPV of early mR was 95 % (19/20 patients) and 72 % (13/18 patients), respectively. False-positive early mR was found in 5/38 patients (13 %) (Table 4b). These five patients were classified as mR (class I) on early PET but reclassified as class II (four patients) or class IV (1 patient) at the late metabolic evaluation. Of the four class II patients, three developed single lesions that did not respond to therapy, and one developed two lesions. For the remaining patient, the analysis showed an mPD with the appearance of three new lesions (two bone lesions and one lung lesion), clearly indicating a rapid progression of the disease that was not responding to therapy.

### Prognostic value of early mR heterogeneity

**Fig. 4:**
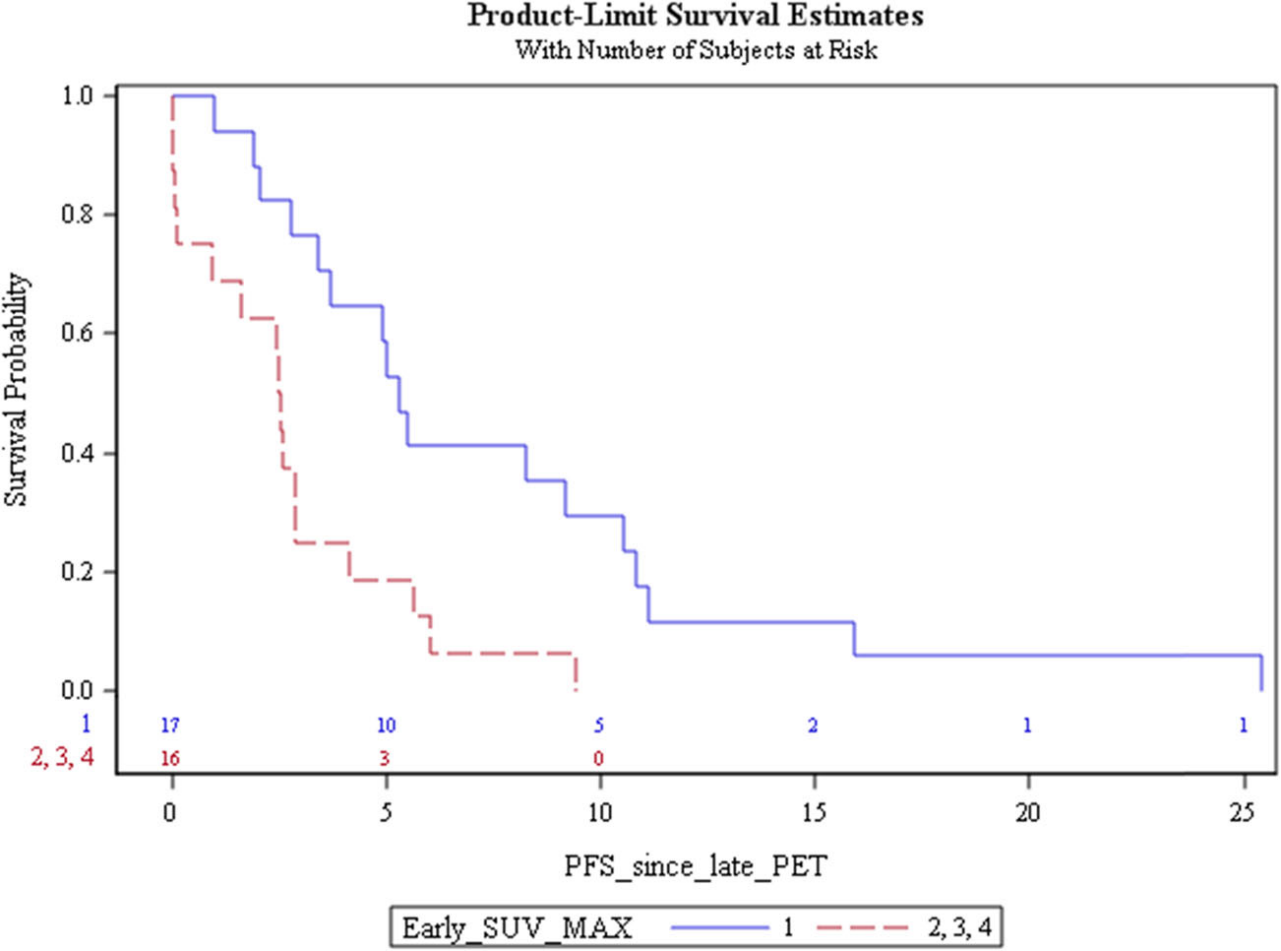
PFS curves estimated by the Kaplan-Meier method according to early homogeneous mR (class I) versus heterogeneous mR (classes II, II, and IV). The difference between the median PFS of early mR patients with a homogeneous response (class I) and non-responder patients with a heterogeneous response (classes II, III, and IV) is statistically significant (*p* = 0.003)

Survival curves revealed a significant difference between median PFS of early mR patients with a homogeneous response (class I) and patients with at least one non-responding lesion: 5.3 months (95 % CI 2.8–10.5) versus 2.5 months (95 % CI 0.1–2.9) [*P* = 0.003, hazard ratio (HR) 0.33 (95 % CI 0.15–0.72)] (Fig. 4). PFS within class II, III, and IV patients seemed similar. No significant difference was observed between median OS curves of early mR patients (class I) and mNR patients (classes II, III, and IV): 12.0 months (95 % CI 8.2–16.6) versus 7.7 months (95 % CI 4.2–12.4) [*P* = 0.27, hazard ratio (HR) 0.68 (95 % CI 0.34–1.37)].

### Analysis of survival curves (PFS) of early and late PET populations

To evaluate whether a patient selection bias occurred, an analysis of survival curves and median PFS was performed for two groups of patients: The first group was composed of patients who had undergone an early but not a late PET (*n* = 40), and the second group was composed of patients who had undergone both an early and a late PET (*n* = 38). With this approach, the aim was to determine whether the group to which a patient belonged could influence his/her survival. The median PFS was 3.0 months (95 % CI, 2.1 to 4.0 months) in the first group of patients (early PET without late PET) and 3.5 months (95 % CI, 2.6 to 5.7 months) in the second group of patients (early and late PET). No statistically significant difference was found between the median PFS of the two groups (p-value: 0.15).

## Discussion

Standard metabolic response assessment using FDG PET-CT is usually performed 8–12 weeks after therapy initiation, together with a morphology-based imaging test [10]. At that time point, FDG PET-CT has been shown in several haematologic and solid tumour types to be a better surrogate for treatment outcome than conventional imaging, in both neo-adjuvant and palliative metastatic settings [5, 21–23]. Standardized patient preparation, imaging, and response reporting (EORTC/PERCIST) criteria have been proposed, allowing the use of PET in multicenter trials [24]. Moreover, as metabolic changes always precede morphological changes, FDG PET-CT allows clinicians to detect treatment resistance and/or response much earlier in the treatment. The tumour shrinkage is less pronounced using targeted drugs, associated with a cytostatic effect, consequently leading to a low sensitivity of the radiological response imaging tools. An early detection of treatment resistance is, however, needed, as it would increase the cost-effectiveness of treatment through better patient selection and reduce needless toxicity, related loss of quality of life, and society costs. Another related issue is the increasing awareness of response heterogeneity, which should be identified early in treatment to provide a basis for response-based treatment adaptation (e.g., by adding surgery or locoregional therapy targeting the resistant lesion or by changing the systemic treatment). The objective of this study was to test FDG PET-CT in a patient cohort with chemoresistant metastatic CRC who were treated with sorafenib combined with capecitabine for early detection of metabolically resistant lesions/patients through correlation with standard metabolic and morphological response assessment (at 6–8 weeks) and patient outcome (OS/PFS). A first observation in this study was that no response fulfilling the RECIST criteria was observed, invalidating its use as a true reference of efficacy in the current study.

As the objective of the study was the early identification of patients with tumour refractory to the drug combination through the characterization of interlesional metabolic response heterogeneity, we developed a descriptive methodology with a 4-class response classification system defined by the presence and proportion of non-responding lesions within the whole body tumour load. The cut-off value for defining non-response with the highest NPV was set to a 15 % decrease of SUV_max_, based on earlier data obtained in comparable patients treated with polychemotherapy [15]. This cut-off is lower than the cut-offs for response detection proposed in EORTC (25 %) and PERCIST (30 %) that are used for standard response assessment later in treatment [18, 19]. Because of this lower cut-off, the probability of unjustified stopping of treatment should be as low as possible. Using such a cut-off in this study, early FDG PET-CT was highly predictive for a metabolic non-response at a later time-point evaluation on both a lesion basis (NPV 85 %) and a patient basis (NPV 95 %). This demonstrates that treatment resistance can already be detected as early as after one cycle. Moreover, treatment resistance at the early PET was related to early treatment failure, as indicated by significantly shorter PFS observed in patients with at least one non-responding lesion in the early PET-CT (median PFS of 2.5 months versus 5.3 months in homogeneous mR patients).

A few reports exist in the literature, but only on early mR prediction in patients with mCRC treated by classic (non-targeted) chemotherapy. All of these studies used higher response cut-off values primarily aimed at detecting mR and not treatment resistance. Bender et al., in a lesion-based analysis, demonstrated that after one cycle of 5-FU chemotherapy, a reduction of FDG uptake occurred in therapy-sensitive metastases – evaluated by morphological imaging for objective response – and a stable or enhanced FDG uptake was indicative of therapy resistance and/or progressive disease [25]. In contrast, Byström et al. did not report a significant correlation after two cycles of chemotherapy between mR and time to progression/OS [26]. Several methodological limitations of that study were reported [27]. First, there were no data regarding the histopathological subgroups and related baseline FDG avidity of the mCRC patients [28]. Second, the size of lesions was not indicated. It is known that in small lesions, the quantitative characterization of tumour metabolism with FDG PET-CT involves the partial volume effect as one of the main sources of error [29]. In the current study, target lesions were defined on the baseline FDG PET-CT according to strict criteria: FDG avidity (higher than two times the normal liver/thoracic aorta uptake) and size (more than 15 mm). More recently, some studies showed that an FDG PET-CT evaluation after a single course of chemotherapy is able to discriminate, with a high NPV, patients unlikely to benefit from the treatment in terms of both tumoural shrinkage and general outcome (PFS/OS) [9, 10, 12].

This study did not observe any signs of a “metabolic flare phenomenon”, previously described in a report by Findlay et al. as a transient metabolic pseudo-progression at 1–2 weeks after the start of chemotherapy in ultimately responding lesions [30]. Such a false-positive non-response early metabolic response during chemotherapy has not been confirmed by later studies. Sources of error in the report by Findlay et al. were the lack of imaging standardization and of patient preparation at that time (e.g., timing between injection and start of PET acquisition ranged from 45 to 99 min; in our study this time lag was fixed at a maximum of 15 min) [30]. The flare phenomenon, in the context of chemotherapy and molecular targeted therapy, seems, therefore, to be more a misinterpretation due to all types of biases than a real biological phenomenon that could be observed in humans with in vivo metabolic imaging [31]. Indeed, only small animal PET studies have reported this phenomenon after chemotherapy; i.e., early (D1-7) transient increase in FDG accumulation due to an inflammatory infiltrate that consists of neutrophils, lymphocytes, and macrophages at the treatment site [32]. A transient early metabolic flare in lesions that ultimately will respond to treatment has been well described in tumours following radiotherapy (due to inflammation) and hormonal therapy (due to a partial agonistic effect) [33–35]. It is expected that new cancer immunotherapies using antibodies, vaccines, and cell-based therapies will probably demonstrate a metabolic flare, thereby impairing the future use of FDG as a tracer for early detection of treatment resistance [36].

In this study, only 1/38 (3 %) patients and 6/124 (5 %) lesions were falsely considered as non-responding on early PET. All of these six early mNR lesions were classified as mSD on the early PET. In four of these six lesions, the false-negative mR was based on the threshold effect (15 % for early vs 25 % for late PET). For the remaining two lesions, no plausible explanation of the postponed metabolic responses was found. These six early mNR lesions had a minor impact on the patient-based response classification. Indeed, a change of patient-based classification occurred in only one patient (a shift from class II at early mR to a class I at late mR).

This study particularly looked at interlesional tumour response heterogeneity and its evolution during treatment. It was found that in all but one patient, tumour heterogeneity persisted during treatment and that in patients with early heterogeneous responses, the number of treatment-resistant lesions increased with time. These results reflect that treatment resistance persists with time or can evolve in a more aggressive disease [37]. The latter observation underscores the negative impact of response heterogeneity on patient outcome, which was also confirmed by the PFS analysis that showed a significant drop in PFS in the presence of at least one resistant lesion (median PFS of 2.5 months versus 5.3 months in homogeneous mR patients). In an earlier report on early FDG PET response assessment in mCRC using polychemotherapy without targeted treatment, a similar drop in PFS was described when the major part of the tumour load was non-responding [6]. In this study, performed with a larger patient cohort and a more potent therapy, it could be shown that even patients with a minor non-responding tumour load on early PET did have impaired prognosis. An ongoing trial using a treatment from the same family as sorafenib (regorafenib) is currently seeking confirmation of these results [8, 38]. That, if granted, will generate subsequent prospective trials assessing the value of early PET to guide treatment adaptation based on the presence and proportion of non-responsive lesions with the aim to impact positively the patient care and health economics.

### Limitations of this study

A potential limitation of the study is that the standard “late” PET was only performed in a subset of patients (*n* = 38) participating in the main trial, which included 94 patients. However, all patients included in the substudy systematically underwent the late PET. To formally exclude a bias through preferential selection of patients for the late PET assessment who were responding to treatment, a comparison of survival (PFS) of the two groups of patients (early versus early and late PET) was performed and did not reveal a significant difference of median PFS between the two groups. As shown in the results section, three centers did not systematically perform a late PET. Only five patients in these centers were included in the late metabolic analysis. These five patients represent 13 % (five out of 38) of all patients included in the late PET analysis. If there was a selection of center bias, it was very limited.

## Conclusion

Early metabolic response assessment using FDG PET-CT after one cycle of a combined sorafenib-capecitabine in patients with chemorefractory mCRC allows, with a high accuracy, the early identification of lesions resistant to treatment. Due to the absence of the so-called “metabolic flare phenomenon” and the very low false-negative rate of patient/lesion, a later time point metabolic response evaluation is not necessary if the early metabolic response assessment has demonstrated treatment resistance.
